# Human Serum Albumin: From Molecular Aspects to Biotechnological Applications

**DOI:** 10.3390/ijms24044081

**Published:** 2023-02-17

**Authors:** Alessandra di Masi

**Affiliations:** Department of Sciences, Section of Biomedical Sciences and Technologies, “Roma Tre” University, 00146 Rome, Italy; alessandra.dimasi@uniroma3.it

Human serum albumin (HSA), the most abundant protein in plasma, is a monomeric multidomain macromolecule that represents the main determinant of plasma oncotic pressure and the principal modulator of fluid distribution between body compartments. HSA is a valued biomarker in many diseases, (i.e., cancer, rheumatoid arthritis, ischemia, obesity, and diabetes) and finds clinical application in the treatment of several pathologies, including shock, trauma, hemorrhage, acute respiratory distress syndrome, hemodialysis, acute liver failure, chronic liver disease, and hypoalbuminemia [1]. In recent years, a novel role of HSA is emerging in the innate immunity [2,3,4,5]. Indeed, HSA acts as a self-defense agent toward *Clostridium difficile* and *Streptococcus pyogenes*, as well as against *Candida albicans* infection by inactivating toxins produced by these pathogens [2,3,4,5,6]. In this Special Issue, original studies on HSA structural, molecular, and functional aspects in health and disease, as well as those related to its biotechnological applications, are covered.

Starting from the structural aspects of HSA capability to bind drugs, Liberi and coworkers [7] reported the structural analysis of Gemfibrozil (GEM) in complex with albumin. GEM is an orally administered lipid-regulating fibrate derivative drug, known under the brand name Lopid^®^. GEM is widely used to treat hypertriglyceridemia and other disorders in the lipid metabolism. Though generally well tolerated, GEM can alter the distribution and the free active concentration of some co-administered drugs, leading to adverse effects [1]. In a paper by Liberi and colleagues, the crystal structure of HSA in complex with GEM has been reported. The elucidation of the molecular interaction between GEM and HSA offered the Authors the possibility to speculate on the development of novel GEM derivatives that can be safely and synergistically co-administered with other drugs, potentially improving its therapeutic efficacy [7].

In addition to being able to bind and transport drugs, HSA displays an extraordinary ligand-binding capacity, serves as the main carrier for fatty acids, recognizes metal ions, and accounts for most of the anti-oxidant capacity of human plasma. Among these pleiotropic functions, De Simone and coworkers [8] widely discussed the esterase, enolase, glucuronidase, and peroxidase intrinsic and metal-dependent (pseudo)-enzymatic activities of HSA. HSA-based catalysis is physiologically relevant as it affects the metabolism of ligands, such as lipids, cholesterol, reactive oxygen and nitrogen species (ROS and RNS, respectively), and drugs. Of note, the catalytic properties of HSA are modulated by allosteric effectors, competitive inhibitors, chemical modifications, pathological conditions, and aging [8]. Interestingly, the Authors highlighted the possible exploitation of HSA enzymatic properties in the “green chemistry” field, by using this protein as a scaffold to produce libraries of catalysts for water decontamination from poisonous agents and environmental contaminants [8].

One of the most important functions of HSA is it capability to transport endogenous and exogenous molecules. This is possible thanks to the fact that this protein possesses nine ligand binding sites (FA1 to FA9). The inherent ligand-binding properties of HSA make it highly desirable for biotechnological applications. In particular, the potential applications of HSA for human cell culture represent by-products of the physico-chemical, biochemical, and cell-specific properties of this protein. The paper by Mishra and Heath [9] is an up-to-date excursus on the structural and biochemical features of HSA in the field of eukaryotic cell culture. HSA ligand-binding capacity is discussed, with particular attention to critical components of cell culture media, such as pyridoxal, riboflavin, and proteins. Of note, the HSA-bound ligands are internalized into eukaryotic cells being essential for optimal cell growth and survival [9]. The Authors highlighted the importance of acquiring even more information on the structural and functional properties of HSA, as they can be exploited by DNA recombinant technologies to design HSA variants characterized by higher affinity for specific ligands, enhanced metal binding properties, or strong ROS scavenging activity [9].

From a biotechnological point of view, HSA represents a promising drug delivery carrier, thanks to the presence of the Cys34 residue and 59 Lys residues that can be used as scaffolds for covalent modifications of drugs. Obara and colleagues developed a Tyr-selective modification technique using the tyrosine click method [10]. By comparing three different protocols, they found that the laccase-catalyzed method can efficiently modify Tyr residue(s) of HSA under mild reaction conditions without inducing oxidative side reactions. Mass spectrometry analyses suggested that Tyr84, Tyr138, and Tyr401 are the main modification sites of HSA [10]. Unlike the conventional Lys residue modification, the Tyr modification method proposed by Obata et al. caused a minimal inhibition of drug binding, thus suggesting that this modification could represent a promising method for covalent drug attachment to HSA [10].

The *N*-terminal site (NTS) of HSA is known as a metal (e.g., Co^2+^, Cu^2+^, Ni^2+^) binding site, displaying a superoxide dismutase-like activity that prevents ROS formation. In a study by Cho and colleagues [11], a peptide derived from the NTS of HSA was designed and synthesized to develop a peptide-based chelate. This peptide was demonstrated to be able to bind to Co^2+^ and to be safe for eucaryotic cells [11]. The Authors concluded that this peptide could be used as a peptide chelate able to bind radioactive isotopes, which are used as drugs or contrast agents in the medical field [11].

A further paper published in this Special Issue focused on the biomedical aspects associated with HSA. The gene encoding for HSA (i.e., *ALB*) is characterized by a significant DNA polymorphism, resulting in phenotypes ranging from the expression of genetic variants (alloalbuminaemia or bisalbuminaemia) to the complete absence of the circulating protein (congenital analbuminaemia). As discussed in the paper by Caridi and colleagues [12], these genetic variants are not associated with disease, neither in the heterozygous nor in the homozygous form. Only the variants resulting in familial dysalbuminaemic hyperthyroxinaemia and hypertriiodothyroninaemia are clinically relevant, as patients are at risk of inappropriate treatment or may have adverse drug effects [12]. For these reasons, drugs that are highly bound to HSA or have a narrow therapeutic index may require therapeutic drug monitoring in these patients.

In conclusion, this Special Issue provided an overview of the biochemical, biomedical, and biotechnological properties of HSA that allow this protein to be still largely exploited for a huge number of applications in several research fields.

## Data Availability

Not applicable.
